# Insight on Photocatalytic and Photoinduced Antimicrobial Properties of ZnO Thin Films Deposited by HiPIMS through Thermal Oxidation

**DOI:** 10.3390/nano12030463

**Published:** 2022-01-28

**Authors:** Endrika Widyastuti, Jue-Liang Hsu, Ying-Chieh Lee

**Affiliations:** 1 Department of Tropical Agriculture and International Cooperation, National Pingtung University of Science & Technology, Pingtung 91201, Taiwan; endrika_w@ub.ac.id; 2 Department of Agricultural Product Technology, Faculty of Agricultural Technology, Universitas Brawijaya, Malang 65145, Indonesia; 3 Department of Biological Science and Technology, National Pingtung University of Science & Technology, Pingtung 91201, Taiwan; jlhsu@mail.npust.edu.tw; 4 Department of Materials Engineering, National Pingtung University of Science & Technology, Pingtung 91201, Taiwan

**Keywords:** thermal oxidation, ZnO whisker, *E. coli*, *S. aureus*, *C. albicans*

## Abstract

Zinc oxide thin films have been developed through thermal oxidation of Zinc thin films grown by high impulse power magnetron sputtering (HiPIMS). The influence of various sputtering power on thin film structural, morphological, photocatalytic, and antimicrobial properties was investigated. X-ray diffraction (XRD) analysis confirmed that the crystalline phase of ZnO thin films consists of a hexagonal wurtzite structure. Increasing the sputtering power will lead to intrinsic stress on thin films that promote whisker formation. In this study, whiskers were successfully developed on the thin films without precursors/catalysts and not thermally treated over the Zn melting point. This finding showed that the film phase structure and morphology are significantly affected by sputtering power. It was found that ZnO thin films exhibit high photocatalytic performance under UV irradiation (89.91%) of methylene blue after 300 min of irradiation. The antimicrobial activity on ZnO thin films showed significant inhibition activity (*p* < 0.05) against *E. coli*, *S. aureus*, and *C. albicans*. However, the whisker formation on ZnO thin films is not accessible to enhance photocatalytic and antimicrobial activity. This study demonstrates that the HiPIMS method through the thermal oxidation process can promote a good performance of ZnO thin films as photocatalyst and antimicrobial agents.

## 1. Introduction

Metal oxide nanomaterials have been found to exhibit superior photocatalysis by the hinge on the interaction of light and solid particles of semiconductors. Among the various transition metal oxides, Zinc Oxide (ZnO) is the most intentionally studied inorganic material with broad applicability to many fields. Its material is acknowledged as the most crucial photocatalyst that strongly resists chemical and photo corrosions [1]. Photocatalysis has been considered a facile, green and cost effective method used for the degradation of dyes [2,3,4], catalytic process [5], Solar cells [6], photodetector application [7], and antibacterial applications [8,9].

Several methods have been introduced to synthesize the ZnO thin films, such as chemical vapor deposition [10], molecular beam epitaxy [11], sol-gel [12], pulsed laser deposition [13], and High Power Impulse Magnetron Sputtering (HiPIMS) [14]. Among these processes, the HiPIMS technique is a potential approach for developing high-quality thin films due to high-density plasmas with excessive quantities of ionized species, high energy transfer functions, low duty cycles, and long pulse off periods that lead to a substantially lower total heat load to the substrate compared with other methods [15,16,17].

Reactive HiPIMS, widely used to prepare Zn compounds to become ZnO thin films, was reported in many publications [18,19,20,21,22,23]. This technique promises the large-scale production of ZnO thin film with high packing density and strong adhesion. Additionally, reactive sputtering benefits controlling thin film structure, composition, and photocatalytic properties due to the ease of modifying the deposition parameters like sputtering pressure, sputtering power, deposition time, and oxygen concentration [24]. Tiron et al. [25] demonstrated ZnO thin film growth using reactive HiPIMS from Zn target resulting in films that exhibit more prominent grain with higher refractive index without post-growth annealing. However, due to the excessive ions sputtered during deposition to the target, the reactive HiPIMS approach is less efficient, lowering the deposition rate and decreasing film thickness [26]. One approach to address this issue utilizes the post thermal oxidation method, which grows a thin layer of oxides on the surface of the substrate. Post thermal oxidation method generates ZnO films from the metallic Zn layers, which takes advantage of its simplicity and noticeable economy of the process [27].

Recently, several studies have reported developing ZnO films using thermal oxidation from sputtered Zn metal films [27,28,29,30,31]. The layered structure of the deposited Zn layers is changed from metal to Zn oxide phase by thermal oxidation, which involves the direct heating of a variety of metal substrates at a temperature in the range of 300–800 °C in the presence of air [32]. The thermal oxidation temperature plays a crucial role in the crystal structure and various ZnO morphology. ZnO films synthesized by this method demonstrated excellent optoelectronic properties that were highly sensitive to the temperature employed during growth synthesis [29,33]. It has to be pointed out that using high-temperature over Zn melting point (419.5 °C) drives to change in the morphology form become ZnO whisker (ZnO nanoneedles) development [34,35]. Instead of thermal stress, the ZnO whisker can be produced with the addition of some precursor/catalyst such as zeolite [36], copper [37,38], gold [39], and chromium [40], as reported earlier. In this study, single-crystal ZnO whiskers on glass substrate were successfully developed using a high sputtering power through thermal oxidation (below Zn melting point) without the addition of precursor/catalyst. This novel development of single-crystal whiskers establishes a new approach for forming single crystals ZnO and several application possibilities. Although thin-film structure and morphology have considerable importance, especially for the ZnO thin film properties, limited published work refers to the development of ZnO through the thermal oxidation of Zn thin films grown by HiPIMS, which are essential for enhancing the photocatalytic activity and antimicrobial capability.

In the last few years, there has been a growing interest in developing antimicrobial coatings based on ZnO nanoparticles [41,42,43] and thin films [44,45,46]. ZnO thin films have shown antibacterial activity on *Escherichia coli* [44,45], *S. aureus* [47], and antifungal activity on *C. albicans* [48]. In contrast, limited work reported a simultaneous antimicrobial activity on ZnO thin films, which allows a wide range of applications. Thus, it is believed that the development of thin films for antimicrobial surfaces to reduce microbial loads on a surface would play an essential role in reinforcing the ZnO application. In this study, ZnO thin films are grown by thermal oxidation of metallic zinc film. Zinc thin films were developed using the HiPIMS method under various sputtering power. The influence of experimental conditions on crystalline behavior, microstructures, photocatalytic and antimicrobial properties of ZnO thin films will be further investigated.

## 2. Materials and Methods

### 2.1. Fabrication of ZnO Thin Films

A pure Zn target (99.99% purity, Ultimate materials Technology Co., Ltd., Hsinchu, Taiwan) with 550 × 125 × 6 mm dimension was used as a target to deposit Zn onto glass substrates using High Power Impulse Magnetron Sputtering (HiPiMS) (Juyong Vacuum Co., Ltd. Tainan, Taiwan). A standard glass substrate (FEA, Microscope slides ground Edges Plain, FEA Industries Inc., Morton, PA, US) was 76 × 25 mm. The substrates were cleaned before using the sonicating and rinsing procedure described in reference [4,9]. The distance between target and substrate was set at 150 mm. In order to investigate the effects of deposition conditions on Zn thin film, various sputtering Powers at 500, 900, and 1500 W were applied. The substrate temperature was controlled at 25 °C, and the flow rate of argon (99.9999%, Xinguang Co., Ltd., Kaohsiung, Taiwan) was fixed at 500 sccm. The Zn film thickness was controlled at about 120–140 nm. The detailed deposition parameter for Zn thin films is given in Table 1.

All thin film samples were thermally oxidized at 400 °C for 2 h to promote ZnO from the metallic Zn thin film layer. The heating rate was 5 °C/min.

### 2.2. Sample Characterization

Film crystallinity was analyzed with X-ray diffraction (XRD; Bruker D8 Advance- AXS Gmbh, Am Studio 2D, Berlin, Germany) using Monochromatic Cu Kα radiation (λ = 1.540598). The voltage and current devices were operated at 40 kV and 40 mA, respectively. The scanning angle 2θ with a value range of 10° ≤ 2θ ≤ 80°, a step size of 0.1° and a time resolution of 5 s per step, was used. The estimated grain size (*D*) of thin films calculated using the full width at half-maximal (FWHM) of the (002) peak using Scherrer’s formula (Equation (1)) [49]:

(1) $$Grain\;size\;\left(D\right)=\frac{K\;\lambda }{\beta \cos\theta }$$

where *D* is the grain size, *λ* is the X-ray wavelength of Cu kα radiation (0.1514 Å), *β* is the width of the peak corrected for the instrumental peak broadening of (002) peak, *θ* is the Bragg’s diffraction angle, and K is correlation factor with value 0.94. The surface roughness of the ZnO thin films with various sputtering power was further measured using atomic force microscopy (AFM, SEIKO SPA400, Seiko Instruments Inc., Chiba, Japan).

Microstructural, morphology, and thickness observations of the thin film cross-sectional were analyzed with field-emission scanning electron microscopy (FE-SEM; Zeiss GeminiSEM 450, Oberkochen, Germany).

Whisker plane-view morphology grown onto glass substrates were made with field-emission transmission electron microscopy (FE-TEM; FEI Tecnai G2 F20, FEI Company—Thermo Fisher Scientific, Hillsboro, OR, USA) integrated with an energy-dispersive spectrophotometer and operating at 200 kV acceleration voltage. A high-order three-beam focused ion beam microscope (FIB) (Hitachi NX2000, Hitachi High-Tech Cooperation, Minato, Tokyo, Japan) was used to prepare the TEM sample. Fast Fourier transform (FFT) was performed using Gatan Software Digital Micrograph software version 3.7.4 (Gatan Inc., Pleasanton, CA, USA).

### 2.3. Optical Properties Characterization

The absorbance and transmittance of ZnO thin films were determined using UV- vis spectrophotometer (UV/Vis, U-3310, Hitachi Ltd., Chiyoda, Tokyo, Japan) in the range wavelength 200–1000 nm and 300–800 nm, respectively. The optical bandgap of the thin films was estimated from the Tauc plots of the optical absorbance. The determination of optical band gap is obtained by Tauc’s equation (Equation (2)) [29]:

(2) $$\alpha \;hv=A\;{\left(hv-E_{g}\right)}^{n}$$

where α is the absorption coefficient, *hν* is the energy of the absorbed photon energy; *A* is a characteristic parameter independent of photon energy, *E_g_* is the optical energy gap, *n* = 2 for allowed direct transition.

The photocatalytic performance of the thin-film samples was evaluated by examining the decolorization of a high-purity methylene blue (MB) (Alfa Aesar; Thermo Fisher Scientific, Lancashire, UK) solution with an initial concentration (*C*_0_) of 10 mg/L. ZnO thin-film samples with size 26 mm × 38 mm were immersed into 10 mL aqueous MB solution then irradiated using UV-A lamp 8 W (UV-A T8; *λ* = 365 nm, Philip, Amsterdam, The Netherlands) for 5 h. The distance between the UV lamp and thin films was kept at 9 cm. The film was immersed in MB solution before UV radiation and kept under dark conditions for 60 min to achieve MB absorption equilibrium on the ZnO layer. From the end of the dark-storage time (*t* = 0 min, *C*_0_), a sample of dye solution was analyzed every 30 min for 5 h (*t* = 30–300 min, *C*_30_ to *C*_300_). The collected sample was analyzed by a UV–VIS spectrophotometer to obtain the absorbance for quantifying the concentration of MB (the maximum absorption wavelength of the dye at 664 nm) [4]. The ratio of the dye concentration that remained (*C*_t_) over the initial dye concentration (*C*_0_) obtained from the absorbance standard curve was evaluated. The degradation performance was calculated following Equation (Equation (3)) [4]:

(3) $$Methylene\;Blue\;Degradation\;\left(\%\right)=\left[1-\left(\frac{C_{t}}{C_{0}}\right)\right]\times 100$$

The rate of the photodegradation of the MB dye on the zinc oxide surface was calculated based on the first-order reaction kinetics equation (Equation (4)) [4]:

(4) $$\ln\frac{C_{t}}{C_{0}}=-kt$$

where *C*_0_ is the initial concentration of MB and *C*_t_ is the MB dye concentration at a particular time (mg/L). In the pseudo-first-order photodegradation kinetics, the ln (*C*_t_/*C*_0_) linear fit slope in the function of time (*t*) plot represents the kinetic constant (*k*) of the photodegradation.

### 2.4. Antimicrobial Activity

The antimicrobial activity of the thin-films materials was assessed using gram-negative *Escherichia coli* ATCC25922, gram-positive bacteria *Staphylococcus aureus* ATCC10234, and pathogenic yeast *Candida albicans* ATCC10234. Bacteria and yeast were streak onto Tryptic Soy Agar (TSB) and Yeast Extract-Peptone-Dextrose (YPD) (1% yeast extract (*w*/*v*), 1% bactopeptone (*w*/*v*), 2% glucose (*w*/*v*)) agar, respectively and incubated at 37 °C for 18–24 h. One single colony of bacteria and yeast were inoculated in 4 mL liquid Tryptic Soy Broth (TSB) and Yeast Extract-Peptone-Dextrose (YPD) (yeast) broth, respectively, and grown aerobically at 37 ± 1 °C on a rotary shaker (160 rpm/min) for 18 h. Subsequently, the microbe cells were collected by centrifuging at 8000 rpm for 5 min, then washed and re-suspended with 10 mM Phosphate Buffer Saline (PBS) solution pH 6. Several dilutions were performed to measure microbe concentration (colony-forming units per mL (CFU/mL). Then the dilution was spread onto agar plates, which were incubated at 37 ± 1 °C for 24 h. The final concentration at 10^5^ and 10^6^ CFU/mL were chosen for the experiment.

**Figure 1:**
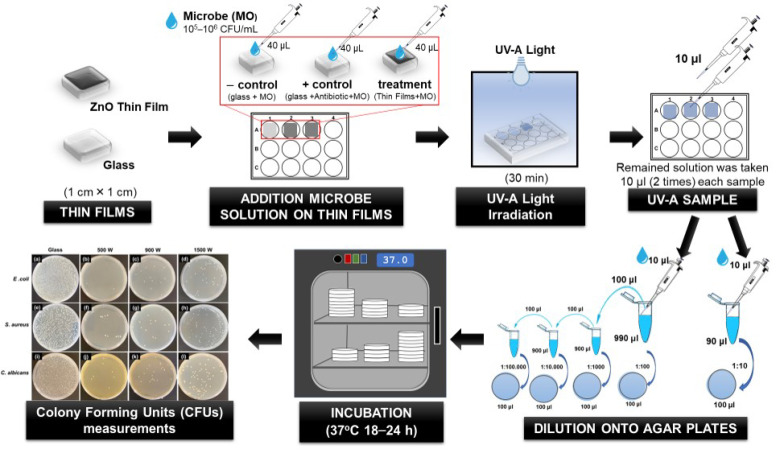
The procedure of ZnO thin-film antimicrobial analysis.

The ZnO thin-film antimicrobial properties were evaluated by the interaction of the microbial cells with the surface of the ZnO thin films under UV-A irradiation, as followed by the previous report [9,47,50] (Figure 1). Thin-film samples of 1 cm × 1 cm, previously sterilized with 70% ethanol for 2 h and rinsed in sterile deionized water, then were placed in 12-weel sterilized cell culture plates (SPL Life Sciences Co., Ltd., Pocheon-si, Gyeonggi-do, Korea). Additionally, substrate (glass with the same size as the thin film) was used as the negative control to ensure that the inhibited microbial growth does not come from UV-A irradiation. Furthermore, three types of antibiotics with different concentrations were applied as a positive control. Kanamycin (100 μg/mL), Ampicillin (100 μg/mL), and Amphotericin-B (10 μg/mL) (Sigma-Aldrich, St. Louis, MO, USA) were used to treat *E. coli*, *S. aureus*, and *C. albicans*, respectively. Before being used, several concentrations of three types of antibiotics were optimized on the percentage of microbe inhibition. The concentration antibiotics were chosen based on the percentage to kill the bacteria >99%. The appropriate antibiotic concentration was added to the microbe solution (10^5^ and 10^6^ CFU/mL), called antibiotic microbe solution.

Subsequently, 40 μL of microbe solution (10^5^ and 10^6^ CFU/mL) were added drop-wise on the glass substrate and thin-film surface. The antibiotic microbe solution was added dropwise on the glass substrate. Then, 12-well plates were closed, sealed, and followed by exposing to UV-A lamp 8 W (UV-A T8; *λ* = 365 nm, Philip, Amsterdam, The Netherlands) for 30 min at room temperature (25 ± 2 °C). The distance between the sample and the UV lamp was approximately 9 cm. All the experiments were conducted in a sterile environment.

After 30 min irradiation with UV-A lamp, 10 μL of the remained solution on glass and thin film surface samples was taken two times then diluted with 90 μL and 990 μL PBS solution. Serial dilution was performed, then spread onto agar plates, which were incubated at 37 °C for 18–24 h. At these incubation times, the number of Colony Forming Units (CFUs) measurements were applied to estimate the number of viable microbe cells that survive after interaction with each thin film during irradiation time [5]. The percentage of microbe inhibition was calculated by the following formula (Equation (5)) [2]:

(5) $$Percentage\;\;Inhibition=1-\left(\frac{CFU\;treated}{CFU\;control}\right)\times 100$$

CFU treated corresponds to the microbe concentration (CFU/mL) after irradiating with UV light, and CFU control is the original microbe concentration (CFU/mL). All tests were carried out in triplicate to verify the results.

### 2.5. Statistical Analysis

The photocatalytic activity data were analyzed using OriginPro version 9.1 software (OriginLab Cooperation, Northampton, MA, USA) and presented as mean ± SD of three replicates. To determine significant group differences in percentage microbial inhibition, one-way analysis of variance (ANOVA) and Post-hoc Tukey HSD tests were performed by using SPSS Statistics version 16 software (SPSS for Windows; IBM Corp., New York, NY, USA). Means were considered statistically significant if *p* < 0.05.

## 3. Results and Discussions

### 3.1. ZnO Structure Analysis

**Figure 2:**
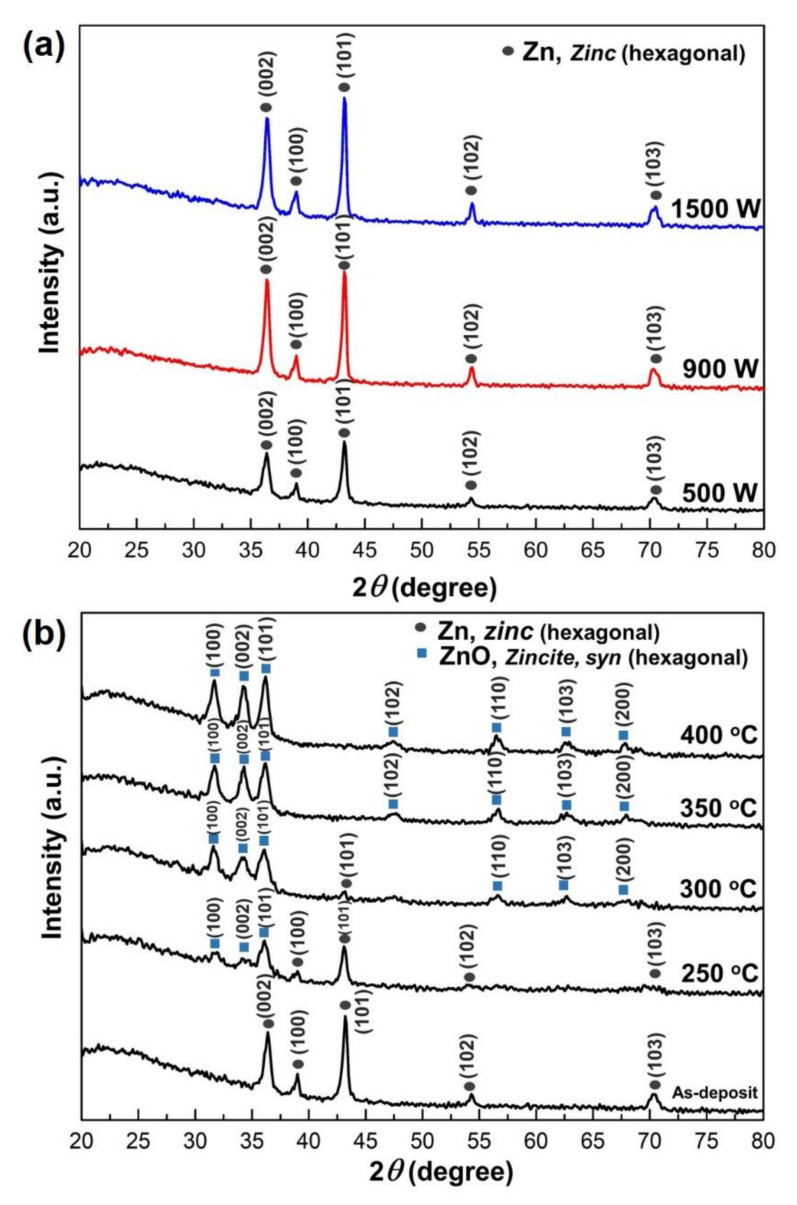
X-ray diffraction (XRD) pattern of Zinc thin films (**a**) As-deposit with various sputtering power and; (**b**) Zn thin films deposit at 500 W under different oxidation temperatures.

The crystal phase of the Zn films on the glass substrate was characterized by X-ray diffraction pattern (XRD) analysis. Figure 2a shows that the as-deposited Zn thin films in various sputtering power are hexagonal structures (JCPDS card 01-087-0713). The Zn crystalline intensity was strongly affected by the sputtering power during deposition. The film grown with the lowest sputtering power (i.e., 500 W) exhibited a low-intensity peak. An order magnitude increase in the Zn peak intensity was achieved when the sputtering power increased. The intense reflection at 2θ = 36.3° and 43.23° indicate that in respective films, the Zn microcrystalline grow preponderantly with the (002) and (101) planes parallel to the substrate surface, respectively. It is well known that increasing sputtering power means the density plasma increases significantly, and large fractions of sputtered atoms are ionized, resulting in high-density films [51].

In order to observe the influence of oxidation temperature on the Zn thin films, the Zn thin films deposited at 500 W were thermally oxidized from 250 to 400 °C, as seen in Figure 2b. After oxidation, zinc is transformed into ZnO (JCPDS card 00-036-01451). The crystalline quality and ZnO orientation showed a strong dependence on temperature parameters during the thermal oxidation process. The maximum oxidation temperature was set at 400 °C, not exceeding the Zn melting point temperature (419.5 °C). Peaks of (100), (102), and (002) orientations of the ZnO wurtzite structure enhance significantly during the thermal oxidation process. As the oxidation temperature increases, the crystallinity of the ZnO thin films can be enhanced, revealed by decreasing the full-width at half maximum (FWHM) of the principal diffraction peak [28]. This result is consistent with previous observations of ZnO films prepared by thermal oxidation of Zn [27,52,53].

**Figure 3:**
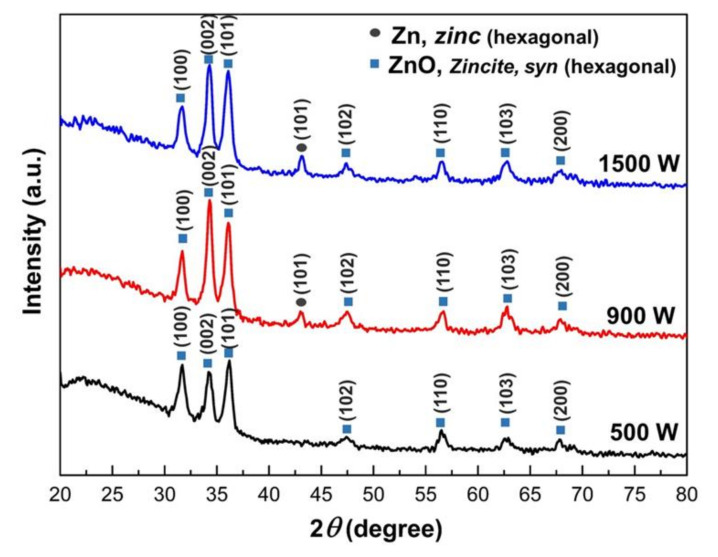
X-ray diffraction (XRD) pattern of Zinc thin films thermally oxidized at 400 °C for 2 h under various sputtering powers.

Thin films with various sputtering power, thermally oxidized at 400 °C for 2 h are shown in Figure 3. All the films in this study show an intense peak at 2θ = ~34.3°, corresponding to ZnO’s (002) planes. It indicates that the films have a preferential growth along the c-axis of the wurtzite unit cell of ZnO. This result was in agreement with previous literature [21]. The ZnO (002) diffraction peak remained a prominent feature for the thin films grown with applied sputtering Power of 500 W to 1500 W. The peak intensity increased with the increasing Zn sputtering power, indicating the high crystalline quality of the formed ZnO films, indicating preferred (002) orientation. The Zn-ZnO transformation on Zn films deposited at 500 W showed complete oxide. However, ZnO with high sputtering power (900 W to 1500 W) was not fully oxide, which exhibited Zn phases. This indicates that the insufficient oxygen for Zn thin films during thermal oxidation is due to film thickness being too much thicker. This will be discussed in microstructure analysis (Section 2.2).

The Zn thin film thickness was maintained ~120 nm at various sputtering powers. However, after Zn thin films were thermally oxidized at 400 °C, the thickness was varied around 146 to 226 nm, as listed in Table 2. The thickness of ZnO thin films seems to increase with increasing sputtering power. It is widely established that plasma or glow discharge forms arise during sputtering through the avalanche ionization of the gases. Thus, the degree of such ionization affects the plasma density. Consequently, as the sputtering power increases, the plasma density will increase the number of sputter ions, giving a higher deposition rate that develops high thickness thin films [23,51].

The (002)- oriented grain size (D) of ZnO thin films can be estimated using the Scherrer equation. It was observed that the thin film grain size would decrease with the increasing sputtering power. The particles are deposited with high energy will promote a high amount of migration particles present in the substrate, and ion bombardment would interfere with crystal growth and prevent the formation of large grains. If there is lower migration when the particles condense on the substrate, the grain size will be larger [18,54]. It could be seen that the grain size of the films decreases from 18.15 nm to 14.12 nm and the root mean square (rms) value of the roughness increases from 25.88 nm to 36.89 nm, when the sputtering power increased from 500 W to 1500 W. By developing ZnO thin films using thermal oxidation methods, the grain size in this study showed lower than ZnO grain size development using reactive HiPIMS that earlier reported [19,23,55]. It is, thus, demonstrated that developing ZnO through thermal oxidation of Zn thin films grown by HiPIMS can promote small grain size. In addition, increasing the sputtering power will decrease the grain size of the crystalline film and the rms roughness of ZnO films.

The variation of the lattice constant of ZnO thin films is described in Table 2. The a-lattice constants were calculated from XRD peak corresponding to (100) plane using the following equation (Equations (6)–(8)) [56]:

(6) $$d_{hkl}=\frac{1}{\sqrt{\frac{4}{3}\;\left(\frac{h^{2}+k^{2}+hk\;}{a^{2}}\right)+\frac{l^{2}}{c^{2}}}}$$

(7) $$a=\frac{\lambda }{2\sin\theta_{\left(100\right)}}\;\sqrt{\frac{4}{3}\left(h^{2}+hl+k^{2}\right)+{\left(\frac{a}{c}\right)}^{2}l^{2}}$$

(8) $$a=\frac{\lambda }{\sqrt{3}\;sin\;\theta_{\left(100\right)}}$$

where *h*, *k*, and *l* are the Miller indices. *d_hkl_* is the lattice spacing determined from Bragg’s equation. The c-lattice constants of the ZnO thin films are determined from the XRD peak corresponding to (002) plane, using the following equation (Equations (9) and (10)) [56]:

(9) $$c=\frac{\lambda }{2\sin\theta_{\left(002\right)}}\;\sqrt{\frac{4}{3}{\left(\frac{c}{a}\right)}^{2}\left(h^{2}+hl+k^{2}\right)+l^{2}}$$

(10) $$c=\frac{\lambda }{\;sin\;\theta_{\left(002\right)}}$$

The *a*- and *c*- lattice constant of the ZnO obtained from the JCPDS card were used as standard with values of 3.250 and 5.207, respectively. The calculated lattice constant of ZnO thin films with different sputtering power is shown in Table 2. ZnO thin films exhibit higher values compared to the standard. According to Ennaceri et al. [56], the thin films presenting higher lattice constant value are subject to tensile strain along the c-axis perpendicular to the substrate surface, which assumes a unit cell tensile along the c-axis direction. The c-lattice constant decreases as a function of increasing sputtering power and eventually stabilize at 1500 W. However, the a-lattice constant increases with increasing sputtering power and its indicating reduction of residual stress in ZnO films. The residual stress involves thermal and intrinsic components, while intrinsic stress could be triggered by the film deposition parameters such as temperature, applied power, and pressure [57]. The intrinsic stress could generate defects like twins, precipitates, disoriented grains, grain boundaries, and microcracks [27]. The various sputtering powers are responsible for such a morphological difference, which will be discussed later. Further study of the microstructure using FE-SEM will be performed to prove this supposition.

### 3.2. ZnO Thin Film Microstructure

**Figure 4:**
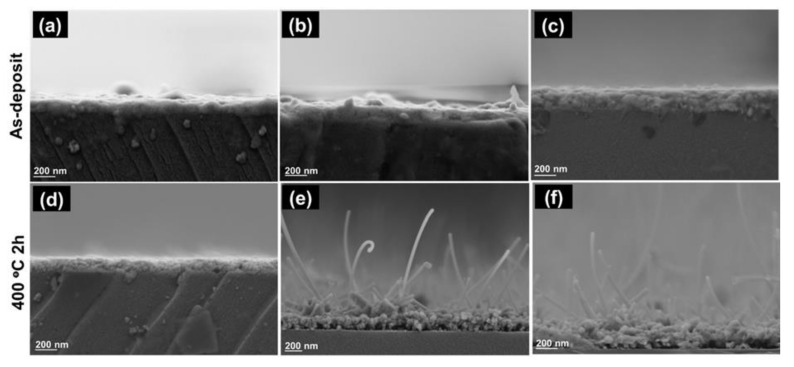
Field Emission Scanning Electron Microscope (FE-SEM) analysis of Zn as-deposited (**a**–**c**) and thermally oxidized (ZnO) (**d**–**f**) thin film with various sputtering power. Cross section imaging of Zn films deposited at: (**a**) 500; (**b**) 900; and; (**c**) 1500 watt and ZnO thin films deposited at: (**d**) 500; (**e**) 900; and; (**f**) 1500 watt.

Field Emission Scanning Electron Microscope (FE-SEM) micrographs examined cross-section imaging of the as-deposited Zn and ZnO thin films with various sputtering power was examined by Field Emission Scanning Electron Microscopes (FE-SEM) micrographs. Figure 4 displays a typical FE-SEM micrograph of as-deposited Zn and ZnO thin films. The morphology of Zn thin films was found to be good homogenous and dense film under various sputtering Power (Figure 4a–c). The thickness of Zn thin films after thermal oxidization at 400 °C seems to increase with the increasing sputtering power, which is found around 146 nm to 226 nm, as mentioned in Table 2.

In addition, various numbers of the whisker were observed on ZnO thin films deposited at 900 and 1500 W, as displayed in Figure 4d–e. The whiskers with diameters around 50 nm appear to grow randomly on ZnO films. The average length of the whiskers was observed around 700–1200 nm, and whisker length and density increase linearity with increasing deposition power. The appearance of whisker could be induced from applied of high sputtering power, which leads to intrinsic stress on thin films and creates some defect or morphological difference on thin films, as mentioned in Section 3.1.

**Figure 5:**
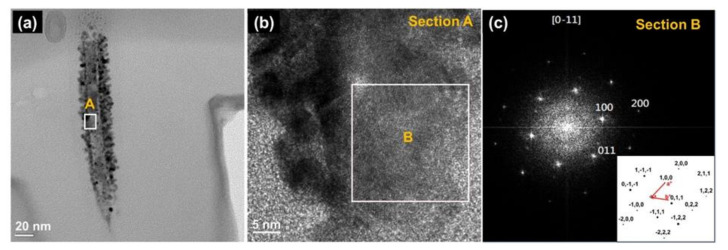
Transmission Electron Microscopes (TEM) micrographs of whisker on ZnO thin films: (**a**) Single whisker; (**b**) High-Resolution TEM (HR-TEM) of Section “A”, and (**c**) Fast Fourier Transform (FFT) of Section “B” corresponding to the [0$\bar{1}$1] beams direction (inset: lattice vectors).

The whisker on ZnO thin films was analyzed using High-resolution Transmission Electron Microscopes (HR-TEM). Figure 5a presents a bright-field TEM image of an individual ZnO whisker, and Figure 5b displays an HR-TEM image of a single column. The Fast Fourier Transform (FFT) image of section B indexed as [0$\bar{1}1$] or [1$\bar{2}$1$\bar{3}$] beams direction, as shown in Figure 5c. The results reveal that the ZnO whisker is a single crystal of wurtzite structure. The (100), (011), and (200) microcrystalline planes of the ZnO wurtzite structure co-existed in the ZnO whisker, and this observation is consistent with the XRD results. It demonstrates a broad range of grain orientations that result in spot patterns with randomly oriented grains. These results were in good agreement with a previous report [58].

Generally, the whisker development on ZnO were confirmed to grow in single crystals due to thermal stress, vapor-liquid-solid (VLS) [36,39], thermal evaporation [34,35] methods or addition of some precursor (catalyst) particle such us zeolite [36], copper [37,38], gold [39], or chromium [40]. The impurities from the precursor will suppress the ZnO film’s grain growth during heat treatment, causing localization of the film stress relief, resulting in whisker formation [37]. In addition, several researchers were reported promoting ZnO whisker from Zn metal [34] and powder [35], pretreated with temperature over the Zn melting point (500–800 °C). It is hypothesized that the partial melting and the evaporation of Zn might exhibit during the initial stage of annealing. These may contribute to the formation and growth of whisker oxide [30]. However, in this study, a ZnO whisker developed on the thin films neither contained the precursor nor was intentionally thermally treated over the melting point. This result supposes that the whisker growth on the ZnO deposit during the thermal oxidation process may come from the high density of Zn during sputtering with high sputtering power (i.e., 900 and 1500 W) that gives higher internal stress. Increasing sputtering power will promote a high density of whiskers during thermal oxidation that will influence the surface roughness of thin films. This result is easy to be understood. The high roughness for both ZnO thin films deposited at 900 and 1500 W come from the whisker, as described in Table 2. Additionally, these findings indicated that the intrinsic stress was predominantly determined by the film grain size, with higher stress relief occurring in layers with larger grain size or thickness. Atoms prefer to nucleate on low-energy surfaces and grow along the orientations corresponding to high-energy surfaces [59].

In addition, the two phases (Zn and ZnO) co-existing on ZnO thin films that develop through thermal oxidation with high sputtering power (900 and 1500 W) may be responsible for the emergence of whiskers. Li et al. [30] reported that a co-existance of Zn and ZnO fine particles and or fine particles consisting of inner core Zn and outer shell ZnO can stimulate the nucleation oxides process during the initial oxidation stage that prevents evaporation of the melting element and preferred growth along a particular direction, thus giving rise to the formation of whisker oxides.

Recently, the development of ZnO whiskers in the form of a thin film [37,38], rod, needle, rugby-like [60], or tetrapod-like whiskers [61] has also been reported. However, a single-crystal substrate, such as sapphire, diamond, or (0001)α-Al_2_O_3_, is required to effectively form single-crystal film or whiskers [62]. In this paper, we report for the first time the growth of single-crystal ZnO whiskers on glass substrate using a high sputtering power through thermal oxidation at 400 °C (below Zn melting point) without the addition of precursor/catalyst. In this study, the novel development of single-crystal whiskers ZnO establishes a new approach for synthesizing single crystals and several application possibilities.

### 3.3. Optical Properties and Band Gap Energy

**Figure 6:**
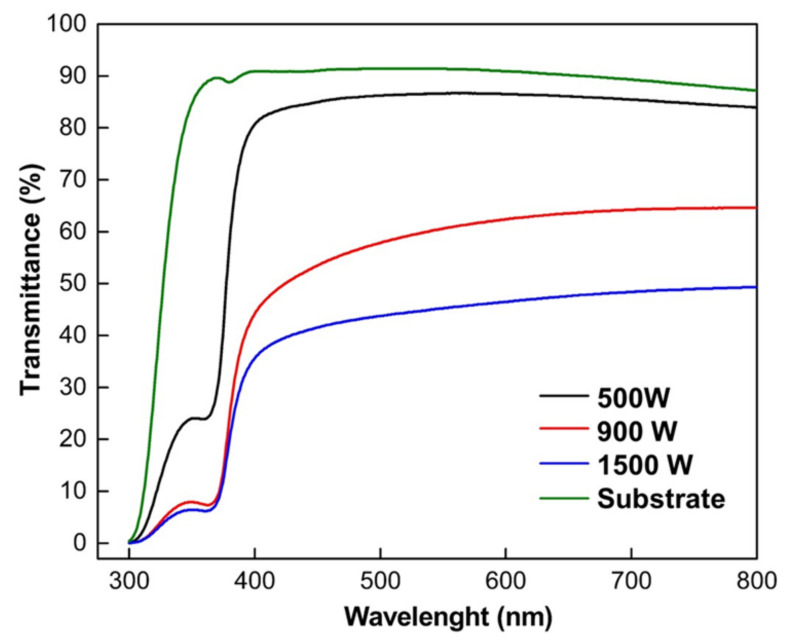
Optical transmittance spectra of ZnO thin films with various deposition sputtering power.

Transmission spectroscopy measurements were made on the ZnO thin films to compare the influence of sputtering power. Transmission spectra as a function of wavelength are illustrated in Figure 6. The ZnO thin film’s optical transmittance of the samples was measured between 300 to 800 nm with glass substrate of the same size as the reference. The average transmittance in the visible range is around 49.3–83.9% for all thin films. The ZnO thin-film transmittance spectra curves gradually shift to a short wavelength as the increasing sputtering power. The optical transmittance spectra of ZnO thin films deposited at 500 W were exceptionally clear and more than 83% in the visible region. This result was in agreement with [21] that reported developing ZnO thin films using reactive-HiPIMS.

In contrast, increasing sputtering power at 900 W to 1500 W decreases film transmittance from 64.6 to 49.3%, as seen in Figure 6. The increasing optical transmittance can be due to enhanced crystallinity [63] and decreased optical scattering caused by densification of film crystallites, thickness, and defects [64]. The lower transmittance in ZnO films with high sputtering power could be described as follows. First, the oxide films formed by oxidation of Zn films deposited in high sputtering power show a dense structure with non-oriented whiskers on the outer surfaces. Understandably, a dense surface structure increases the possibility of the reflection of incident radiation. Second, both ZnO samples deposited at 900 and 1500 W have the presence of the (101) Zn crystal peak, as shown on XRD patterns (Figure 3), implying the Zn films do not fully oxidize. Incorporation of metallic Zn connected to high absorption in the metallic layer, related to the lower oxygen incorporation and the resulting high density of absorption centers developed within the gap oxygen vacancies as well as the morphological effects [29]. These phenomena resulted in poor light transmission in ZnO samples deposited in high power.

**Figure 7:**
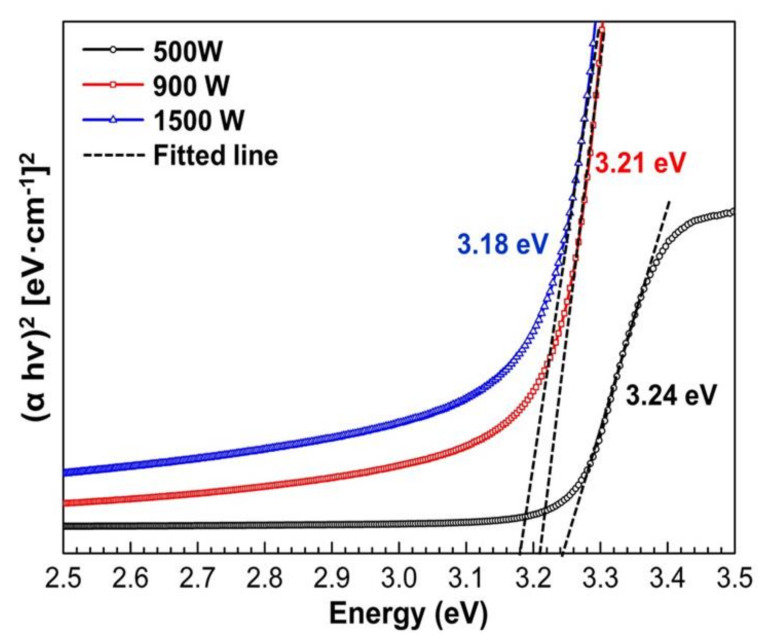
The plot of (α hv)^2^ versus photon energy (hv) of the ZnO thin films with various sputtering power.

The absorption spectra were used to calculate ZnO thin film energy bandgap (Eg). The optical band gap of ZnO thin films was calculated by plotting the graph of the incident photon energy (hv) versus (αhv)^2^. The energy band of ZnO film with various sputtering power is shown in Figure 7. The bandgap values of ZnO thin films are 3.24, 3.21, and 3.18 eV for 500, 900, and 1500 W, respectively, and the corresponding wavelength exits in the UV region. It also can be observed that the energy band gap of prepared ZnO thin films is lower than that bulk ZnO 3.3 eV [65]. However, the bandgap shift becomes more evident with low sputtering power due to the fully crystalline ZnO structure (as illustrated in Figure 4). The increasing bandgap indicated reduced visible light absorption. Consequently, the light’s wavelength is crucial for photocatalytic efficiency. The absorption spectra imply that UV light is a good source for the photocatalytic activity of ZnO. The energy bandgap is monotonically decreasing with increasing sputtering power. Rusu et al. [28] reported that small grain size in thin films would affect the electrostatic potential in the grain boundaries and attributed to decreasing energy bandgap. As explained in Table 2, increasing sputtering power will decrease the ZnO grain size, which leads to the lowering energy bandgap. In addition, the lower value of Eg may be also due to the greater density of donor states near the conduction band, determined by the oxygen vacancies on the thin films. This results in good agreement with XRD analysis that shows ZnO thin films deposited in high sputtering power (900 and 1500 W) have a Zn crystal peak that implies the Zn films do not fully oxidize.

### 3.4. Photocatalytic Activity

**Figure 8:**
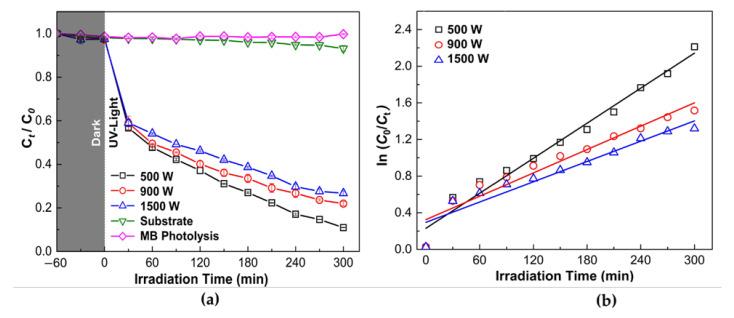
Photodegradation of Methylene Blue (MB) solution by photocatalytic reaction with ZnO thin films under UV-light (*λ* = 365 nm) irradiation and (**a**) Degradation rate (**b**) Kinetic curves (first-order reaction) of thin film samples with MB under various deposition power. The experimental condition is: MB concentration (*C*_0_) = 10 mg/L, T = 25 ± 2 °C. Data were expressed as mean ± standard deviation (*n* = 3).

The ZnO thin film photocatalytic activity was determined by the degradation of 10 mg/L of MB solution under UV-A light irradiation with 300 min irradiation time, as shown in Figure 8a. Prior to use, all samples were immersed in MB aqueous solution in dark condition for 60 min for the evaluation degree of the adsorption of MB on the beaker wall and the sample surface. The adsorption is reported in grey color in Figure 8a. It can be observed that ZnO thin-film samples exhibited fast adsorption in the dark. The degradation efficiency of MB solution increases with increasing irradiation time. It was found that ZnO thin films demonstrate considerably high photocatalytic performance compared to the substrate and MB photolysis under UV irradiation. The MB degradation percentage for ZnO sample deposit at 500, 900, and 1500 W were 89.91, 78.80, and 73.98%, respectively. The highest degradation efficiency was found for ZnO 500 W. Therefore, the degradation efficiency of the ZnO thin films reduces by increasing sputtering power.

In addition, the linear relationship between ln (*C*_0_/*C_t_*) and reaction time was used to study the photocatalytic process further, as shown in Figure 8b. The photocatalytic reaction process was fitted with the pseudo-first-order reaction kinetics for all thin-film samples. The reaction constants (*k*) of the ZnO thin films deposited at 500, 900, and 1500 W were 6.37 × 10^−3^, 4.25 × 10^−3^, and 3.69 × 10^−3^ min^−1^, respectively, showing a higher rate of degradation for ZnO thin film deposited at 500 W. As observed from Figure 7, the reaction constants (k) gradually decreased with an increase in the sputtering power.

Lowering of photocatalytic activity and reaction rate (*k*) on ZnO thin films could attribute to the existence of (101) Zn phase on ZnO thin films that deposited in high power (900 and 1500 W), which also decrease the optical transmittance, as seen in Figure 2b. It is well known that defect states (e.g., oxygen vacancies) in the thin-film crystal structure contributed to the lowering photocatalytic activity. According to Fouad et al. [66], incomplete ZnO growth will affect poor photocatalytic activity. Moreover, complete ZnO growth increases the specific surface area due to the rise in the proportion of atoms on the semiconductor surface, followed by a change in particle surface, featuring surface defects. Semiconducting photocatalysts mainly depend upon separating the photo-generated hole–electron pairs and migration of electrons from the photocatalyst into the organic pollutants entrapped inside oxygen vacancy defects on the surface [67].

Generally, appearing whisker/nanoneedles on a thin-film surface will promote a larger surface area, and the effective surface should be an order magnitude larger than original thin films [68]. However, the presence of whisker formation on the ZnO thin film surface could not promote increasing photocatalytic activity and reaction rate. The results emphasized that the crystallite phase is essential to promote high photocatalytic activity since the appearing large surface area on the ZnO whisker is not accessible for reactant molecules. This result was in good agreement with Xie et al. [60]. Particle morphologies on which crystal faces are exposed affect the photocatalytic activity of the ZnO sample. Furthermore, the particle morphology does not directly relate to the photocatalytic activity and the surface area on different morphology ZnO particles [61]. It is noted that ZnO photocatalytic activity strongly depends on some specific exposed crystal phases [69].

Several works have reported the photocatalytic activity of ZnO thin films with different preparation, as listed in Table 3. However, limited published work reported the photocatalytic activity of pure ZnO thin films developed using HiPIMS methods. At the same irradiation time, the photocatalytic activity obtained in this research is higher than in previous research [65,70], even with lower thin film thickness. The photocatalytic activity of thin films depends on the following factor: (1) thin film profile such as thickness, morphology, element composition, etc., (2) photolysis such as type, concentration, and volume, (3) light intensity, and (4) equilibrium state of thin-film and photolysis. This result demonstrated that developing ZnO thin films using HiPIMS through thermal oxidation can improve the high photocatalytic activity of thin films.

In this study, the photocatalytic of ZnO thin films depends on the crystallite phase than the film morphology. It could be noted that the whisker formation on ZnO thin films is not accessible to enhance photocatalytic activity. Films grown with low sputtering power and relatively thin films demonstrate good photocatalytic activity due to high optical quality and compact morphology. These studies indicate that the HiPIMS power plays crucial parameters in obtaining the high photocatalytic performance of ZnO thin films. Further analysis was established to investigate the correlation between the photocatalytic activity and antimicrobial activity of ZnO thin films with various sputtering power.

### 3.5. Antimicrobial Activity

**Figure 9:**
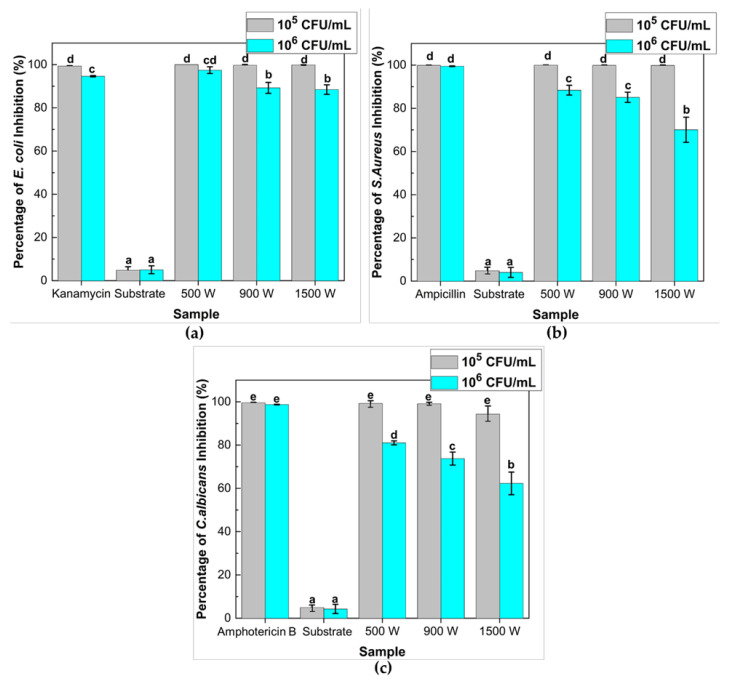
Effects of various deposition power of ZnO thin films on the inhibition growth of microbes. Percentage inhibition of (**a**) *E. coli* ATCC25922; (**b**) *S. aureus* ATCC25923; and (**c**) *C. albicans* ATCC10234 after 30 min UV-A irradiation treatment under different microbial concentrations. Viable microbe recovered after 24 h of incubation at 37 °C, then colonies (CFU/mL) were counted, and the percentage of inhibition was calculated and plotted against various deposition powers of thin films. Antibiotic (Kanamycin (100 μg/mL); ampicillin (100 μg/mL); and Amphotericin B (10 μg/mL)) and substrate (glass) were used as positive and negative control, respectively. Means denoted by a different letter indicated significant differences between treatments (*p* < 0.05; Tukey HSD test). Data were expressed as mean ± standard deviation (*n* = 3).

ZnO thin films were examined for their antimicrobial activities against the gram-negative (*E. coli* ATCC25922) and positive (*S. aureus* ATCC25923) bacterial strain as well as pathogenic yeast (*C. albicans* ATCC10234). The results were also compared to those caused by a glass substrate and antibiotics, as presented in Figure 9. In order to analyze the antimicrobial effectiveness, the initial concentration of microbe was varied at 10^5^ CFU/mL and 10^6^ CFU/mL. To ensure that the inhibited microbial growth does not come from the effect of UV-A irradiation, the microbial suspension was irradiated under UV-A lamp without any ZnO thin film (glass substrate). The negative controls, i.e., glass substrate under UV-light, exhibit a low inhibitory effect on the growth of *E. coli*, *S. aureus*, and *C. albicans* (less than 5%), while, in the presence of the ZnO film, there was a high inhibitory effect of microbe after 30 min of irradiation.

Alternately, the groups treated with antibiotics as positive controls showed high antibacterial inhibition against the three types of pathogenic microbes. A preliminary in vitro assay was conducted to determine the proper antibiotic type and concentration that gave a high percentage inhibition (>99%) to the *E. coli*, *S. aureus*, and *C. albicans*. These tests concluded that the three types of antibiotic kanamycin, Ampicillin, and amphotericin-B were chosen to treat *E. coli*, *S. aureus*, and *C. albicans*, respectively.

All ZnO thin films at 10^5^ CFU/mL microbial concentration exhibited a significant antimicrobial effect (*p* < 0.05) with average percentage inhibition around 99% in the presence of UV light. When the microbial concentration increased (10^6^ CFU/mL), the data showed that the inhibition percentage for *E. coli*, *S. aureus*, and *C. albicans* diminished remarkably, with deposition power increasing after 30 min of UV irradiation. According to Adams et al. [41], the antimicrobial activity of the ZnO sample could be attributed to several parameters, including surface chemistry and morphology, light intensity, and microorganism concentration.

The ZnO thin films deposited at 500 W manifested high inhibition of around 97.43, 88.39, and 81.01% against *E. coli*, *S. aureus*, and *C. albicans*. This result could associate with the crystalline phase and morphology of ZnO thin film, as described in Section 2.4. The complete ZnO growth will increase specific surface area due to increased percentage atoms on the semiconductor surface, promoting high photocatalytic and antimicrobial activity. In addition, the antimicrobial activity of ZnO thin films also depends on the crystallite phase rather than the film morphology, which revealed that the whisker formation on ZnO thin films is less possible to enhance microbial inhibition. It proved an excellent linear correlation between antimicrobial inhibition and photocatalytic activity on ZnO thin films.

**Figure 10:**
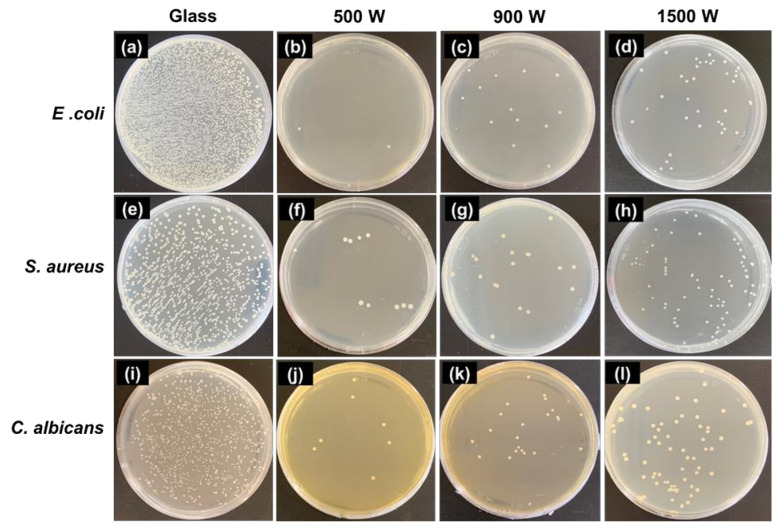
The culture growth on pure agar after the photocatalytic treatment of a microbial suspension (10^5^ CFU/mL) on the zinc oxide thin films fabricated under various deposition power (under 30 min of UV-A). (**a**–**d**) gram-negative (*E. coli* ATCC25922); (**e**–**h**) gram-positive (*S. aureus* ATCC25923); and (**i**–**l**) pathogenic fungi (*C. albicans* ATCC10234). The initial microbial concentration was 10^6^ CFU/mL. Substrate (glass) was used as a control, recovered after 24 h of incubation at 37 °C.

The photographs of the viable microbe colonies (white spot) on the control and samples are shown in Figure 10. The antimicrobial results suggest that all the samples could restrain bacterial growth. In particular, ZnO 500 W shows higher antimicrobial activity than ZnO 900 and 1500 W. Moreover, the maximum antimicrobial activity is achieved as 100 and 97.43% in activation over 500 W for initial microbe concentration 10^5^ and 10^6^ CFU/mL, respectively.

**Figure 11:**
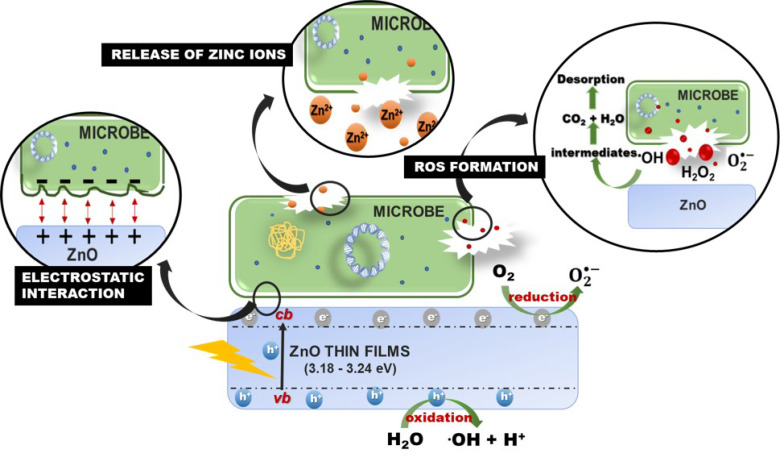
Schematic representation antimicrobial effect on ZnO thin films.

The antimicrobial effect on ZnO thin films against three-types of pathogenic microbe could be attributed to the intrinsic antimicrobial properties of Zn^2+^ ions released by ZnO [73,74], the destabilization of microbial membranes upon electrostatic interaction between ZnO and microbe cell wall [75], and the reactive oxygen species (ROS) formation by UV light radiation [76] (Figure 11). It has been found that the release of Zn^+2^ antimicrobial ions in the media containing the microorganisms is a possible antimicrobial mechanism [73,74,77]. In addition, Devirgiliis et al. [78] reported Zn^2+^ could accumulate fast in both vacuoles and zincosomes of fungi, which serve as an essential cellular protective mechanism to balancing the zinc excess and deficiency. The contribution of Zn^2+^ to the antimicrobial effectiveness of ZnO nanoparticles in the dark condition is minor due to the low concentrations of solubilized Zn species released from ZnO dissolution [79,80]. Furthermore, Zn^2+^ release would be limited by an intrinsic ZnO property, mentioned earlier, which is ZnO stability in water. The insolubility of ZnO impedes the distribution of zinc ions into the medium and thus limits this antimicrobial effect [81].

Pasquet et al. [74] demonstrated that the Zn^2+^ release mechanism is influenced by two major parameters: (i) the physicochemical properties of the particles, including porosity, concentration, particle size, and morphology. (ii) The medium’s chemistry: the pH, UV illumination, exposure time, and presence of additional elements. In this study, the Zn^2+^ release could come from the UV-illumination process. It was observed that there is no inhibition zone found on the agar during dark conditions. In contrast, the UV irradiated sample for 30 min showed an inhibition zone with a diameter range of 16.87–18.75 mm (Figure S1 and Table S1). However, the contribution of the soluble zinc species in this experiment was not measured due to insufficient sample volume.

Furthermore, it has been observed that the interaction of ZnO with E. coli could affect cell wall disintegration, resulting in the collapse of the bacteria membrane, altered morphology, and intracellular material release [75,82]. In contrast to the earlier reports, several investigations have indicated the presence of ROS as the main responsible for the antimicrobial activity of ZnO during the characteristic wavelength of light absorption [76,83], which promotes the oxidation of cellular structures altering the bacteria permeability while promoting their disintegration [84].

ZnO is a semiconductor thin film that has a bandgap around 3.2 eV. Electrons will be shifting from the valence band (vb) to the conduction band (cb) of thin films when the energy photon is higher than the ZnO bandgap (E ≥ Eg). The vb and cb of ZnO fulfill the requirement for the formation of highly reactive hydroxyl radicals (^•^OH) and superoxide radicals ($O_{2}^{•-})$ on the catalyst’s surface [85]. Photoexcited electron-hole pairs ($h_{vb}^{+}$) interact with adsorbed electron donors and acceptors (H_2_O and O_2_) on the ZnO surface to generate highly reactive hydroxyl radicals (^•^OH) (Equation (12)) and superoxide anions radical ($O_{2}^{•-}$) (Equation (13)) during the photocatalytic process. Furthermore, dissolved oxygen molecules are converted into superoxide radicals ($O_{2}^{•-})$, and react with H^+^ to form hydrogen peroxide anions (Equation (14)), which then collide with electrons to produce hydrogen peroxide anions (Equation (15)). In addition, H_2_O_2_ molecules were produced by the reaction of hydrogen ions and hydrogen peroxide anions (Equation (16)).

The ROS formation of hydroxyl radical (^•^ OH) and superoxide and superoxide ($O_{2}^{•-})$ are considered to disintegrate the microbial cell into CO_2_, H_2_O, and other harmless substances via numerous chain redox reactions (Equations (16) and (17)) [42,86,87]. However, H_2_O_2_ could penetrate the cells and destroy the microbe [88]. The production of H_2_O_2_ primarily lies on ZnO’s surface, which results in a large number of oxygen species on the surface that have higher antimicrobial activity [89]. The equations for ROS formation in the ZnO thin films are summarized below [90]:

(11) $$ZnO+hv\to ZnO+e_{cb}^{-}+h_{vb}^{+}$$

(12) $$h_{vb}^{+}+H_{2}O\to \;•OH+H^{+}$$

(13) $$e_{cb}^{-}+O_{2}\to O_{2}^{•-}$$

(14) $$O_{2}^{•-}+H^{+}\to HO_{2}^{•}$$

(15) $$HO_{2}^{•}+HO_{2}^{•}\to H_{2}O_{2}+O_{2}$$

(16) $$HO_{2}^{•}+H^{+}+e_{cb}^{-}\to H_{2}O_{2}$$

(17) $$H_{2}O_{2}+O_{2}^{•-}\to •OH+OH^{-}+O_{2}$$

(18) $$•OH+microbial\;cell\to \;Intermediates\to \;CO_{2}+H_{2}O$$

ROS formation is responsible for the various antimicrobial activity mechanisms, including microbial surface absorption, the formation of electron/hole pairs, their reaction of generated pairs with oxygen/water, and diverse intermediates [76,91]. Srinivinas et al. [92] also reported that the stress induced by ROS might alter microbe structure, resulting in the loss of its selective function and energy (ATP) production that affects DNA replication.

ZnO thin films were shown as antifungal activity, decreasing membrane integrity and overall low enzymatic activity in *C. albicans* [46]. In addition, the antifungal activity of ZnO nanoparticles was reported by He et al. [93]. It showed that ZnO nanoparticles could inhibit conidiophores of *P. expansum* and deformity fungal hyphae of *B. cinerea* [93]. This might be related to the abnormal accumulation of nucleic acids and carbohydrates, whereas ZnO may inhibit cell function and result in excessive nucleic acid level. Alvarez-Peral et al. [94] reported that an increase in nucleic acid might be interpreted as a stress response of fungal hyphae, and likewise, an increase in carbohydrates may be related to the fungi self-protection mechanism from ZnO nanoparticles.

In this study, *E. coli* has shown significantly higher susceptibility (91.69%) to ZnO thin films compared to *S. aureus* (81.17%) and *C. albicans* (72.35%) when treated at 10^6^ CFU/mL microbial concentration. This condition has also been reported in ZnO nanoparticles [95,96]. According to Applerot et al. [96], the presence of intracellular antioxidants such as carotenoid pigments in the inner surface of the bacteria will encourage oxidant resistance, as well as the existence of strong detoxification agents like antioxidant enzymes (catalase) that is one of the primary causes of the increased resistance of *S. aureus* to ZnO.

Numerous research efforts have investigated the antimicrobial activity of ZnO thin films fabricated using different methods, as listed in Table 4. In a similar condition, the antimicrobial activity of ZnO thin films that develop using HiPIMS with post-thermal oxidation methods showed higher inhibition activity than those reported previously [44]. Meanwhile, increasing film thickness seems a promising technique to promote antimicrobial activity. According to Carvalho et al. [45], the ideal thin film thickness for antimicrobial activity is around 200–600 nm.

These results indicate that the sputtering power of ZnO thin films plays an essential role in achieving high photocatalytic performance and antimicrobial activity. Furthermore, sputtering power at 500 W through thermal oxidation could provide a complete ZnO crystal phase thin film with good compactness and higher antimicrobial activity. The existence of ZnO whisker on ZnO thin films when sputtering power increases cannot enhance both the photocatalytic and antimicrobial activity on ZnO thin films. Therefore, this study demonstrates that the HiPIMS method through the thermal oxidation process can promote a good performance of ZnO thin films as photocatalysts and antimicrobial agents against pathogenic bacteria and fungi.

## 4. Conclusions

ZnO thin films were successfully fabricated through thermal oxidation of Zn thin films grown by the HiPIMS method. ZnO thin films consist of a hexagonal wurtzite structure. Sputtering power played a role in influencing the structure, morphology, optical and photocatalytic properties of ZnO thin films. The increasing sputtering power through thermal oxidation will lead to intrinsic stress on thin films that promote whisker formation outwardly as well as the addition of precursor/catalyst. This novel growth of whiskers offers a new method for forming single-crystals of ZnO whiskers and several application possibilities. Furthermore, the optical transmittance and energy bandgap decreased with increasing sputtering power. The whisker formation on ZnO thin films is not accessible to enhance photocatalytic and antimicrobial activity. It proved an excellent linear correlation between antimicrobial inhibition and photocatalytic activity on ZnO thin films. ZnO thin films with low sputtering power could promote high photocatalytic and antimicrobial activity. This study demonstrates that the HiPIMS method through the thermal oxidation process can promote a good performance of ZnO thin films as photocatalyst and antimicrobial material. It is believed that the development of high-quality ZnO thin films for coating surfaces with antimicrobial properties could generate possibilities for photocatalytic disinfection.

## Figures and Tables

**Table 1:** Deposition parameters of Zn Thin films.

Parameter	Zn500	Zn900	Zn1500
Sputtering Power (watt)	500	900	1500
Argon flows (sccm)	500	500	500
Base Pressure (torr)	2.5 × 10^−5^	2.5 × 10^−5^	2.5 × 10^−5^
Working Pressure (torr)	3.6 × 10^−3^	3.6 × 10^−3^	3.6 × 10^−3^
Voltage (Volt)	561	652	755
Current (A)	0.73	1.38	1.74
Deposition Rate (nm/min)	141	236	310

**Table 2:** Structural properties of thermal oxidized Zn film grown by HiPIMS.

Sample	Thickness (nm)	Grain Size (*D*) (nm)	Roughness * (*rms*) (nm)	Lattice Parameter	
				*a* = *b* (Å)_(100)_	*c* (Å)_(002)_
Zn 500 W	146	18.155	34.25	3.261	5.225
Zn 900 W	147.65	15.602	44.51	3.263	5.220
Zn 1500 W	225.50	14.117	46.62	3.265	5.227

* Results obtained from AFM analysis. a-Lattice constant (100) plane calculated from JCPDS, a = b = 3.250 c-Lattice constant (002) plane calculated from JCPDS, c = 5.207.

**Table 3:** Photocatalytic activity of ZnO thin films prepared with various methods.

Fabrication	Thickness (nm)	Irradiation Time (min)	Photolysis (Concentration)	Photocatalytic Activity (%)	Ref.
MOCVD	250	300	Dye (Orange II) (3.50 mg/L) *	23	[68]
	910			50	
DC-MS	400	180	MO (5 mg/L)	73.4	[65]
Sol-gel	500	180	MB (3.20 mg/L) *	70.34	[70]
Sol-gel	218	240	MB (3.20 mg/L) *	77	[44]
ALD	30	240	MB (4.78 mg/L) *	~53 **	[71]
SILAR	1870	300	R6G (5 mg/L)	87	[72]
ILGR	100	80	MB (1.60 mg/L) *	~58 **	[56]
HiPIMS, thermal oxidation	140	180 240 300	MB (10 mg/L)	73.69 83.68 89.90	This Work

* calculated from Molar to mg/L. ** calculated from Ct/Co. MOCVD (Metal-organic chemical vapor deposition); SILAR (simple ionic layer adsorption reaction); ILGR (Ion layer gas reaction); MO (Methyl Orange); MB (methylene blue); R6G (Rhodamine6G).

**Table 4:** Antimicrobial activity of ZnO thin films prepared with various methods.

Thin Films (Thickness)	Initial Microbe Concentration (CFU/mL)	Irradiation Time (min)	Microbial Inhibition (%)			Ref.
			*E. coli*	*S. aureus*	*C. albicans*	
Sol-gel (218 nm)	2.3 × 10^6^	30	94.17	-	-	[44]
	2.3 × 10^6^	60	99.50	-	-	
	2.3 × 10^5^	30	99.53	-	-	
	2.3 × 10^5^	60	100	-	-	
DC-MS (200–600 nm)	8 × 10^7^ *	35	45 **	-	-	[45]
DC-MS (148 nm)	~4.9 × 10^7^	480	-	-	4.08 **	[46]
(655 nm)	~4.9 × 10^7^	480	-	-	53.06	
DC-MS (650 nm)	1 × 10^8^ *	1500	-	-	68	[48]
RF-MS (400 nm)	4 × 10^8^ *	15	-	66.27 **	-	[47]
HiPIMS (140 nm)	1 × 10^5^	30	100	99.98	99.20	This work
	1 × 10^6^	30	97.43	88.39	81.01	

* CFU/mL was calculated from optical density (OD) (1 OD= 8 × 10^8^ CFU/mL). ** Microbial inhibition was calculated from Equation (15).

## Data Availability

Data is contained within the article.

## Supplementary Materials

The following supporting information can be downloaded at: https://www.mdpi.com/article/10.3390/nano12030463/s1, Figure S1: Effect of *E. coli* inactivation by ZnO thin film deposit in different sputtering power; (a) dark condition and (b) 30 min UV-A irradiation after 24 h of incubation at 37 °C; Table S1: Comparative analysis of the average zone inhibition diameters of ZnO thin films deposited in different sputtering power on *E. coli*.
