# Supplementary material for: Insight on Photocatalytic and Photoinduced Antimicrobial Properties of ZnO Thin Films Deposited by HiPIMS through Thermal Oxidation
Source: Nanomaterials (Basel). 2022 Jan 28;12(3):463. doi: 10.3390/nano12030463 (PMC8838953; doi:10.3390/nano12030463)
Supplement: Supplementary file 1 [file nanomaterials-12-00463-s001.zip › nanomaterials-1565845-supplementary.pdf]

Supplementary Material

S1. Antimicrobial activity during dark and UV-A treatment

The effect of *E.Coli* inactivation by ZnO thin film during dark condition and 30 min UV-A irradiation (as shows on **Figure S1**) also the average zone inhibition diameters (**Table S1**) was analyzed using agar diffusion technique. The antibacterial activity was determined based on an inhibition zone. The **Figure S1** showed that during dark condition, there are no inhibition zone found on the agar. In contrast, the sample that have UV irradiated for 30 min showed inhibition zone with diameter range 16.87-18.75 mm. No zone of inhibition was observed for the negative control (glass). The highest mean of zone inhibition (18.75 mm) is recorded for ZnO deposited at 500 W. This result in agreement with antimicrobial activity result on Figure 9 (manuscript). In addition, Pasquet et al. [9] demonstrated that the Zn^{2+} release mechanism is influenced by two major parameters: (i) the physicochemical properties of the particles, including porosity, concentration, particle size, and morphology. (ii) The medium's chemistry: the pH, UV illumination, exposure time, and presence of additional elements. In this study, the Zn^{2+} antimicrobial ions release could come from the UV-illumination process. However, the contribution of the soluble zinc species in this experiment was not measured due to insufficient sample volume.

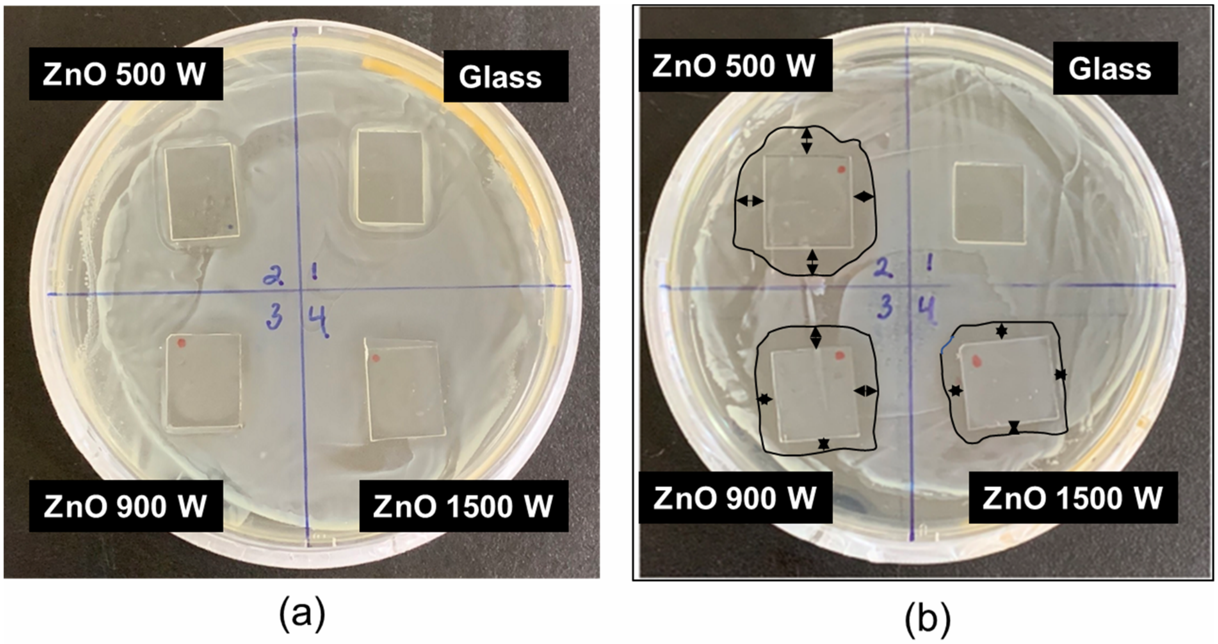

Figure S1. Effect of *E.Coli* inactivation by ZnO thin film deposit in different sputtering power; (a) dark condition and (b) 30 min UV-A irradiation after 24 h of incubation at 37°C.

Table S1. Comparative analysis of the average zone inhibition diameters of ZnO thin films deposited in different sputtering power on *E.Coli*

Condition	Samples	Inhibition Zone Diameter (mm)
Dark	Glass	0
	ZnO 500W	0
	ZnO 900W	0
	ZnO 1500W	0
UV-A irradiation (30 min)	Glass	0
	ZnO 500W	18.75
	ZnO 900W	17.50
	ZnO 1500W	16.87
